# Genetic characteristics and targeted treatments of primary bladder and urachal adenocarcinomas: a systematic review with pooled descriptive genomic analyses

**DOI:** 10.1007/s10555-026-10332-3

**Published:** 2026-05-01

**Authors:** Bálint Dér, Melinda Váradi, Nikolett Nagy, Andras Kubik, Gladell P. Paner, Gopa Iyer, Nadine T. Gaisa, Sara E. Wobker, Richard Bambury, Bas W. G. van Rhijn, Hikmat Al-Ahmadie, Péter Nyirády, Henning Reis, Tibor Szarvas

**Affiliations:** 1 Department of Urology, Semmelweis University, Üllői Út 78/B. 1082, Budapest, Hungary; 2 Departments of Pathology and Surgery, Section of Urology, University of Chicago, Chicago, IL USA; 3 Department of Medicine, Memorial Sloan Kettering Cancer Center, New York, NY USA; 4 Institute of Pathology, University Hospital RWTH Aachen, RWTH Aachen University, Aachen, Germany; 5 Institute of Pathology, University Hospital Ulm, University of Ulm, Ulm, Germany; 6 Department of Pathology and Laboratory Medicine, University of North Carolina, Chapel Hill, NC USA; 7 Medical Oncology, Cork University Hospital, Cork, Ireland; 8 Department of Surgical Oncology (Urology), Netherlands Cancer Institute - Antoni Van Leeuwenhoek Hospital, Amsterdam, The Netherlands; 9 Department of Urology, Caritas St. Josef Medical Center, University of Regensburg, Regensburg, Germany; 10 Institute of Pathology, University of Duisburg-Essen, Essen, Germany; 11 Dept. of Urology, University Duisburg-Essen, Hufelandstr 55, Essen, 45147 Germany

**Keywords:** Urachal cancer, Primary bladder adenocarcinoma, Genetics, Targeted therapy, Precision medicine

## Abstract

Adenocarcinomas are rare histological subtypes of bladder cancer mainly presenting as primary bladder adenocarcinomas (PBACs), or urachal carcinomas (UrCs). The lack of standardized, evidence-based clinical recommendations for therapy results in individualized treatment decisions. These personalized treatments may be tailored based on molecular analyses to target driver mutations. The genetic background of UrC and PBAC has been increasingly explored through molecular analysis of relatively small case series. Similarly, experiences with targeted treatments are available from individual case reports and single institution case series. Consequently, currently available genetic and targeted treatment data are fragmented, hindering a comprehensive overview and firm conclusions. Therefore, we aimed to catalogue and interpret recent molecular genetic insights and targeted therapeutic experiences in UrC and PBAC in order to provide a basis for improved clinical decision-making. In this review, we screened online databases with search terms selective for genetic and targeted therapeutic data in UrC and PBAC. Utilizing the extracted data, we identified mutational frequencies, created OncoPrints, investigated signaling pathways, and evaluated treatment approaches guided by precision medicine. Our summary of genetic findings and targeted therapy strategies are intended to support off-label options for UrC and PBAC.

**Supplementary Information:**

The online version contains supplementary material available at 10.1007/s10555-026-10332-3.

## Introduction

More than 90% of bladder cancer cases are urothelial carcinomas (UC) [1]. Invasive UC may exhibit a glandular phenotype further classified as UC with glandular differentiation (UCg). Even if the invasive tumor shows complete glandular differentiation, the presence of any UC component, invasive or in situ (CIS), is sufficient to diagnose UCg based on current WHO 2022 criteria [2]. However, pure adenocarcinomas of the bladder (without urothelial components) are rare and represent less than 2% of all bladder cancers [3]. These tumors can arise in the bladder and are designated as primary bladder adenocarcinomas (PBAC) or may originate from the urachal remnant identified as urachal carcinoma (UrC) [4–6].

Prospective clinical trials in bladder cancer often exclude rare histological types such as PBAC and UrC, resulting in a lack of high-level evidence and established systemic therapeutic recommendations; **this is particularly concerning because these histological subtypes are known to have distinct prognosis and treatment outcomes [**7**]**. Some case reports and single center case series reported activity of chemotherapy treatments used in colorectal carcinoma (CRC) combining 5-fluorouracil with platinum therapy in UrC[8, 9]. However, as large prospective trials for these tumor types are difficult to conduct, chemotherapeutic strategies that need to be tested in such trials will likely remain at low levels of evidence. In this context, rational molecularly informed targeted treatment approaches represent a promising approach for UrC and PBAC [10]. Since many targeted therapies do not directly target the altered driver gene itself, but rather other components of the affected pathway, understanding these regulatory pathways are essential for rational treatment planning. The genetic background of UrC and PBAC is increasingly being explored in studies that typically include a low number (n = 5–20) of cases and focus on a single tumor type, which limits detailed comparisons [10–12].

A comprehensive overview of the molecular genetic background of PBAC and UrC is necessary for multiple reasons. First, considering that the surgical treatments of UrC and PBAC differs—radical cystectomy is recommended for PBAC [13], while partial cystectomy with the excision of median umbilical ligament and umbilicus is the preferred treatment for UrC—the differential diagnosis of these histologically similar tumor types is important but challenging, highlighting an unmet clinical need [14]. Therefore, molecular analyses might serve as diagnostic biomarkers in the differential diagnostic process. Second, in order to apply targeted treatments, it is crucial to define the potential driver alterations and signaling pathways that are most frequently affected in UrC and PBAC. This could provide practical information for the selection of the most appropriate targeted gene panel.

In addition to the detection of potentially targetable alterations, it is also important to know whether the identified drugs have ever been administered for the treatment of the given tumor entity. For this, a targeted therapy drug catalogue with details on the genetic alteration as well as on therapy efficacy may provide valuable information for patients and clinicians for off-label precision oncology decisions.

Therefore, in the present study we aimed to provide a comprehensive catalogue of genetic alterations/affected molecular pathways and examples of targeted therapies utilized, in order to support the currently challenging therapeutic decision-making for UrC and PBAC.

## Methods

We conducted a literature search **for a systematic review with pooled descriptive genomic analyses** on 31 st December 2023 on PubMed, Web of Science, Embase, Scopus and Cochrane online (Prospero registration Nr. CRD42022374155). The following search terms were used *((urachal OR urachus) AND (cancer OR carcinoma OR tumor OR tumor)) AND (mutation OR sequencing OR "genetic alteration" OR NGS OR "targeted therapy" OR "targeted treatment" OR "precision medicine" OR "precision oncology")* and for PBAC related publications *((urinary bladder OR primary bladder) AND (adenocarcinoma)) AND (mutation OR sequencing OR "genetic alteration" OR NGS OR "targeted therapy" OR "targeted treatment" OR "precision medicine" OR "precision oncology")*. **Inter-rater agreement was assessed using Cohen’s kappa (Κ) statistics, with values interpreted according to standard guidelines to evaluate the consistency between reviewers. B.D. and M.V. performed screening for the UrC cohort (Cohen’s Kappa = 0.8347); B.D. and A.K. for the PBAC studies (Cohen’s Kappa = 0.9853)**.

We excluded meta-analyses, book chapters, reviews without original data and publications in languages other than English. Selected publications had to include mutational and/or targeted treatment data on an individual patient level. If congress abstracts and publications represented the same dataset, only publications were included. We extracted histological data and if more than one histological type was specified per case, it has been listed as mixed histology. Data on gene fusions was only available from the publications by Necchi, et al. and Cigliola et al. [15, 16]. From PBAC-related publications, malignancies resulting from secondary causes (*e.g.* schistosomiasis, BK virus infection, or malignancy arising after cystoplasty) have been excluded. All included publications are listed in Table S1 [10–12, 15–52]. For UrC, cases with non-adenocarcinoma histology have been collected and analyzed separately from the adenocarcinoma cases included in Table S2. We collected genetic data of UCg as well from the selected publications.

OncoPrint figures have been created using PyOncoPrint [53]. **Genetic data by PCR, Sanger-sequencing, and next generational sequencing have been all collected**. If a gene had multiple mutations, we only plotted a single alteration on OncoPrint (we selected the mutations in this order: deletion, splicing, followed by truncated, frameshifts, nonsense or missense mutations). Unedited data is displayed in Table S2 and multiple mutations in the same gene are highlighted. Mutational frequencies were calculated as all identified mutations per all cases assessed for that particular gene.

To illustrate overlapping and different mutational characteristics between different tumor types (UrC, PBAC, CRC, UC, UCg), we used Venn diagrams in Fig. 2**.** generated at https://bioinformatics.psb.ugent.be/webtools/Venn/.

For Fig. 2. CRC and UC TCGA II (2017) datasets (PanCancer Atlas [54]) were assessed by the cBioPortal [55–57] based on the summarized frequencies of SNVs and CNVs (CRC: https://www.cbioportal.org/study/summary?id=coadread_tcga_pan_can_atlas_2018, UC: https://www.cbioportal.org/study/summary?id=blca_tcga_pan_can_atlas_2018). Oncogenic signaling pathways were selected based on PanCancer TCGA dataset [58] and PathwayMapper visual tool [59] and were created with BioRender.com (Der, B. (2025) (https://BioRender.com/h75f210, https://BioRender.com/q73s936, https://BioRender.com/ u94o889). The overall percentage for affected pathways per tumor entity was calculated based on the number of patients with at least one mutation in the pathway divided by the total number of investigated patients (even if not every gene was sequenced from the signaling pathway). All the affected pathways have been plotted on **Fig. S3***.* from TCGA PanCanAtlas. The schematic figures on Fig. 3*.* were based on the BioRender template of Lei et al. [60].

For the molecular alterations with potential predictive value for immune checkpoint inhibitor (ICI) therapy [61], *e.g.* MSI (microsatellite instability), TMB (tumor mutational burden) and PD-L1 expression, we performed an additional screening of the selected publications. TMB-high tumors were defined as > 10 mutations/megabase. In addition, we collected IHC data on β-catenin and molecular data on APC, given the major relevance of similar histopathological and molecular patterns of UrC and CRC.

## Results

### Genomic comparisons of UrC, PBAC, UCg

**Fig. 1:**
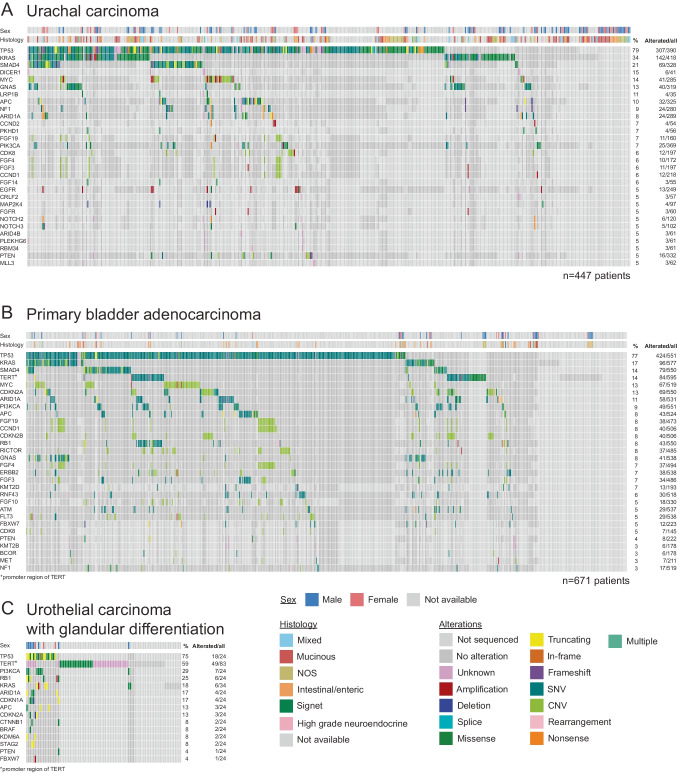
Overview of the genetic alterations in rare adenocarcinomas of urinary bladder. A–C) OncoPrints highlighting mutations in top 30 (urachal and primary bladder adenocarcinomas, **A** and **B**, respectively) and top 15 (urothelial carcinoma with glandular differentiation, **C**) most frequently affected genes.

To gain a deeper understanding of the genetic background of rare bladder adenocarcinomas, we conducted a structured literature search with search terms aiming to identify molecular characteristics (Fig. S1). 28 UrC and 17 PBAC publications were selected for data extraction. Genetic data were available for 447 UrC and 671 PBAC patients. The full tables are shown in Table S2. Although our focus in this study was adenocarcinomas, we also collected genetic data from UrCs with non-adenocarcinoma histologies (Table S2/ UrC non-ADC). These data were only utilized for comparisons with UrC adenocarcinomas but not for further analysis. OncoPrints of top 30 most frequently mutated genes were plotted for UrC and PBAC (Fig. 1A–B). The top 10 mutated genes in UrC were *TP53* (79%), *KRAS* (34%), *SMAD4* (21%), *DICER1* (15%), *MYC* (14%), *GNAS* (13%), *LRP1B* (11%), *APC* (10%), *NF1* (9%), and *ARID1A* (8%). The top 10 frequently affected genes in PBAC were *TP53* (77%), *KRAS* (17%), *SMAD4* (14%), *TERT* (coding (0/21, 0%) and promoter region (84/595, 14%, Fig. 1B), all together (84/616, 14%)), *MYC* (13%), *CDKN2A* (13%), *ARID1A* (11%), *PI3KCA* (9%), *APC* (8%) and *FGF19* (8%). In the nine non-adenocarcinoma UrC cases one neuroendocrine tumor carried *MAP2K1*, *MAP2K4* and *PTPRD* mutations, otherwise *TP53* and *TERT* promoter mutations were the most prevalent (50% and 40%, respectively) followed by *KRAS* in 20% of assessed cases (Table S2/UrC_non-ADC).

To collect genetic data on UCg, we rescreened the same pool of publications. Five papers included a total of 93 UCg patients with their respective genetic data. In this cohort, 15 genes carried mutations that are plotted on the OncoPrint of UCg (Fig. 1C). Top 10 mutated genes in UCg were *TP53* (75%), *TERT* promoter (59%), *PI3KCA* (29%), *RB1* (25%), *KRAS* (18%), *ARID1A* (17%), *CDKN1A* (17%), *APC* (13%), *CDKN2A* (13%) and *CTNNB1* (8%).

Notably, we found many cell membrane receptor genes to be mutated, which are potential molecular targets for personalized therapy. In UrC, these include *NOTCH2* (5%), *NOTCH3* (5%), and *EGFR* (5%); in PBAC, *ERBB2* (7%), *FLT3* (5%), and *MET* (3%). All alterations were bioinformatically detected amplifications (Fig. S2).

**Fig. 2:**
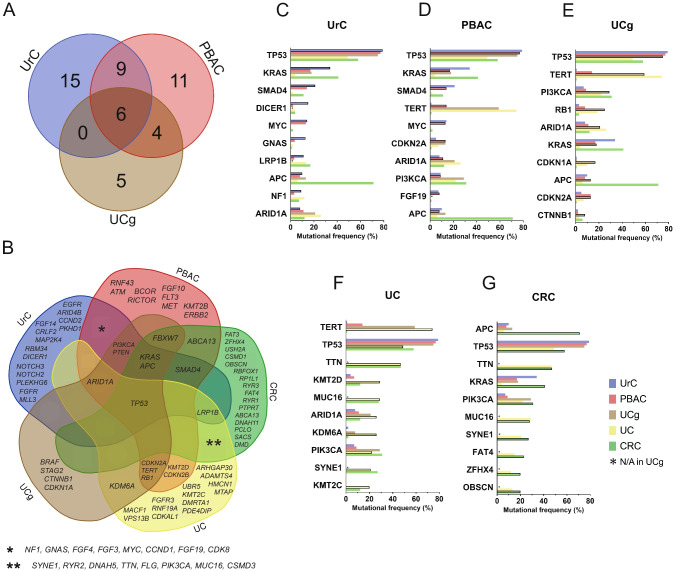
Comparison of the genetic features of bladder malignancies and colorectal carcinoma. **A**) Venn-diagram showing common and unique frequently affected genes of urachal cancer (UrC), primary bladder adenocarcinoma (PBAC) and urothelial carcinoma with glandular differentiation (UCg). Detailed list of genes is displayed in panel **B**) Comparison of the genetic features of glandular bladder malignancies and urothelial carcinoma (UC) and colorectal carcinoma (CRC). Analysis was carried out on the top 30 (UrC, PBAC) and top 15 (UCg) most frequently mutated genes. **C**–**G**) Comparison of the top 10 most frequently mutated genes of each malignancy to other analyzed cancer types. Columns representing the selected malignancy are marked with boarders. If columns are not displayed that implies 0% mutational rate unless differentiate with asterisks when sequencing data of the given gene is not available

Next, we analyzed the overlapping alterations of UrC and PBAC (Fig. 2A) and found a considerable similarity between the two tumor types with 15 of their top 30 most frequently affected genes overlapping (*TP53*, *KRAS*, *SMAD4*, *MYC*, *GNAS*, *APC*, *NF1*, *ARID1A*, *FGF4*, *PI3KCA*, *CCND1*, *FGF19*, *FGF3*, *PTEN,* and *CDK8*). Six of them have been among the top 10 most frequently mutated genes of both tumor types (*TP53, KRAS*, *SMAD4, MYC*, *APC,* and *ARID1A*). When also considering the frequently affected genes of UCg, six of the top genes (*TP53, KRAS*, *PI3KCA*, *ARID1A, APC,* and *PTEN*) were overlapping between all three tumor types (Fig. 2A). UrC and UCg showed no further similarities in their mutational patterns, but PBAC and UCg had four additional genes (*RB1*, *CDKN2A*, *FBXW7* and *TERT* promoter) that are among the top frequently affected ones making the mutational profile of UCg 2/3 overlapping with PBAC, raising the possibility that some of the tumors that are reported as PPAC may represent UCg (see discussion).

### Genomic comparisons with CRC and UC

Following the initial comparison of UrC and PBAC, we extended the scope of our analysis by including CRC and UC genetic alterations. CRC has been suggested to have similar genetic features to UrC [62, 63]. UC was included since it is the most common histological type of bladder cancer. In addition, systemic therapy selection in UrC is often guided by protocols used in either for CRC or UC. As TERT promoter mutations have a high prevalence in UC (74%) and UCg (59%, Fig. 1C) **with differential diagnostic implications, and potential influence on the therapeutic sensitivity of UC to chemo- and immunotherapy**, we highlighted this additional feature in our dataset [64, 65]. Among the five tumor types (UrC, PBAC, UCg, CRC and UC), the mutational profile of CRC was the most distinct with 16 unique genes of the top 30 most frequently altered genes, whereas UCg has only four genes that are not shared with other malignancies (Fig. 2B). In Fig. 2C–F, the top 10 most altered genes of each malignancy are plotted in comparison to the alteration frequencies in the other four tumor types.

*TP53* is the most frequently altered gene in PBAC, UrC and UCg, and the second most affected gene in UC and CRC. The frequency of *DICER1* mutation is uniquely elevated and seems to be selective for UrC. *KMT2D* and *KDM6A* mutations are characteristic for UC. *APC* alterations are the most frequent mutations in CRC (73% compared to only 6–13% in all other assessed tumor types). *PIK3CA* alterations occurred with similar frequency in UC, UCg and CRC (22–31%), but were less frequent in UrC and PBAC (7–9%). In contrast, *MYC* amplifications and *GNAS* alterations were characteristic for both UrC and PBAC but not for the other entities. KRAS mutations were present in 18–41% of UrC, PBAC, UCg and CRC but were sporadic in UC. In contrast, *TTN*, *MUC16* and *SYNE1* were frequently affected in UC and CRC but not in UrC and PBAC. *LRP1B and GNAS* may be useful to differentiate UrC from PBAC. In addition, *TERT* promoter mutation frequency is much higher (> 50%) in UC and UCg (68%) compared to the other three entities.

*RB1* mutation rate is higher in urothelial carcinomas (UC and UCg) compared to the other tumor types. *ARID1A* and *TERT* promoter are predominantly affected in UC and UCg. *TTN*, *MUC16* and *SYNE1* show high frequencies in both UC and CRC but not in UrC or PBAC.

Additionally, one study assessed the *MET* exon 14 skipping alteration at the RNA level in PBAC and UrC, finding a frequency of ~ 10% (2/22 UrC; 1/10 PBAC) of cases [10].

In summary, alterations in *PIK3CA* are rare but *MYC* amplification is more frequent in UrC and PBAC compared to CRC and UCg, and therefore may have a differential diagnostic value. The alterations of *TERT* promoter may help to differentiate UCg from UrC and PBAC. *APC, TTN, MUC16 and SYNE1* may be useful to differentiate PBAC and UrC from CRC. Alterations in *RB1* may differentiate between UCg *vs.* UrC and PBAC. Finally, *DICER1* mutation may help to differentiate between UrC *vs.* PBAC, UC or CRC.

### Molecular characteristics predicting efficacy of ICI treatment

The PD-1 inhibitor pembrolizumab was the first drug to be approved as a tumor-agnostic treatment for unresectable or metastatic solid tumors bearing DNA MMR (mismatch repair) deficiency, MSI-high or high TMB and progression after at least one systemic treatment [66, 67]. TMB, MSI, MMR and PD-L1 are known predictive markers of ICI response [68, 69]. In addition, patients with UrC and PBAC have been treated with ICI and selected cases demonstrated remarkable responses [11, 25]. Although the intensity of PD-L1 expression is affected by several factors [70] and its predictive value for ICI is limited, it is used to support treatment decisions regarding the potential use of ICIs in certain clinical settings [71]. To estimate the prevalence of PD-L1 positivity (as reported in the publications) and MSI status of UrC and PBAC, we extended our data extraction on full text papers containing data on these molecular characteristics. In UrC, 16% (16/102) of patients showed PD-L1 positivity with various antibodies and methods, while PBAC showed a PD-L1 positivity rate of 7% (4/56) (Table S3). High TMB (> 10 mut/Mb) was found in 5% (7/140) of UrC and in 12% (18/148) of PBAC cases. MSI-high status was present in 4–7% of UrC and ~ 1% of PBAC (Table S3).

### Molecular patterns of Wnt signaling

Wnt signaling pathway alterations are hallmarks of tumorigenesis in CRC [72]. As CRC and UrC share a similar morphology, we also analyzed alterations in this signaling pathway [38]. We therefore gathered additional data on the protein expression of nuclear β-catenin by IHC and *APC*-gene alterations from the reviewed papers (Table S3). Nuclear localization of β-catenin was present in ~ 20% of UrC and ~ 10% in PBAC cases. APC – an element of Wnt signaling regulating the degradation of β-catenin – was affected in ~ 10% of both UrC and PBAC cases. To sum up, altered β-catenin localization detected by IHC and *APC* mutations were present in both tumor types, with β-catenin seemingly being affected at higher rates in UrC. However, these alterations showed a much lower incidence compared to CRC, where the nuclear localization of β-catenin was present in the majority (64%) of primary tumors [73].

### Analysis of the oncogenic signaling pathways in UrC and PBAC

**Fig. 3:**
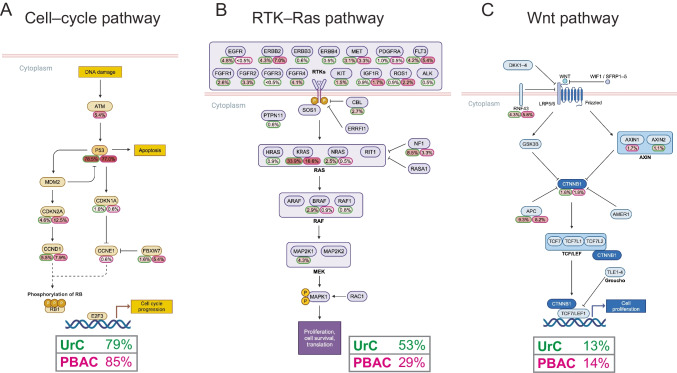
Overview of selected signaling pathways with frequent mutations of urachal carcinoma and primary bladder adenocarcinoma. **A–C)** Selected signaling pathways (cell-cycle, receptor tyrosine-kinase (RTK)–Ras and Wnt pathways) based on Pancancer TCGA dataset [58] and PathwayMapper visual tool [59]. Created in BioRender. Der, B. (2025) (https://BioRender.com/h75f210, https://BioRender.com/ q73s936, https://BioRender.com/ u94o889). Mutational frequencies in UrC are highlighted with green and PBAC with magenta colors for each gene and for the whole pathway (bottom). The calculation of mutation rates of a pathways (boxes) is described in the methods

To understand how the individually altered genes impact complex cellular processes such as apoptosis, cell growth and cell-cycle regulation, we integrated the collected genetic data with a curated selection of oncologically relevant signaling pathways [58]. The top 30 most frequently mutated genes in UrC, PBAC and UCg were involved in the regulation of chromatin structure, cell cycle, DNA replication, cell division, signal transduction of proliferation pathways in addition to growth factors. Eight signaling pathways were found to be affected according to the Pancancer TCGA pathway definitions (Fig. S3**) **[58]. Based on their frequent involvement, we highlighted the receptor tyrosine-kinase (RTK)–Ras [74], Wnt [75] and cell-cycle pathways [76] with their functional features (Fig. 3). As expected from the high rate of mutated *TP53* in both UrC and PBAC (78.5% and 77.0%, respectively), the cell-cycle pathway proved to be highly affected along with the alterations of cyclin-dependent regulatory kinases and effector proteins (*CDKN1A, CDKN1B, CCND1, CCNE1*) (Fig. 3A). Notably, the RTK pathway (Fig. 3B) harbored multiple amplifications in pharmacologically targetable membrane receptors, like *ERBB2* and *MET* in both tumor types (*ERBB2*: UrC: 4.3%, PBAC: 7.0%, *MET*: UrC: 3.1%, PBAC: 3.3%). The mutations of the small G-protein coding *KRAS* gene—a central element of the RTK pathway—were frequent in both UrC (33.9%) and PBAC (16.6%). As a new targeted treatment for patients with the *KRAS*^G12C^ mutation has been approved for lung cancer^77^, this mutation is of significant clinical interest. Therefore, we conducted an additional analysis focusing on *KRAS*^G12C^ mutations and found that *KRAS*^G12C^ mutations rarely occur both in UrC (1/291) and in PBAC (1/214) (Table S2/KRAS G12C).

Since the Wnt pathway is a key player in the oncogenesis of CRC, we highlighted its features as well (Fig. 3C). The intracellular elements such as β-catenin (UrC: 1.6%, PBAC: 1.8%) and its regulatory proteins (*AXIN1*: PBAC: 1.7%, *AXIN2*: UrC: 1.1%, *APC*: UrC: 9.3%, PBAC: 8.2%) were found to be rarely affected. In addition, *RNF43* was the only affected membrane localized protein of the pathway (UrC: 4.3%, PBAC: 5.8%). Pathways that did not carry any mutations from the PanCanAtlas, like the Hippo and the NRF2 pathways were not plotted.

### Targeted treatment

The detailed genetic overview provides theoretical clues for the selection of potentially effective targeted treatments (Figs. 1, 2 and 3). Nonetheless, due to a lack of clinical trials for UrC and PBAC, the collection of published results can synthesize evidence to facilitate off-label clinical decision-making. To identify all published cases (including individual patient data from case studies and series) and to analyze the response rate of targeted treatments, we collected all published targeted treatments from the literature and found data for 29 UrC and 7 PBAC patients from 19 and 5 articles, respectively (Table S4).

For UrC both immune checkpoint and tyrosine kinase inhibitors showed similar objective response rates (ORR, 〜20%) and considerable disease control rates (DCR, > 75%) (Table 1). Of note, all seven UrC patients with high TMB or MSI-high had stable disease on various ICI treatments. In addition, PD-L1 immunostaining was reported to be positive in two UrC patients, both with stable disease identified by radiographic response.

**Table 1:** Summary of published targeted treatment attempts of urachal cancer and of primary bladder adenocarcinoma based on our systematic literature search

**A) Urachal Carcinoma**									
**Target**	**Drug name**	**Molecular target**	**Age (y)**	**Sex**	**Rad. response**	**Resp. duration**	**Survival (OS)**	**Year**	**Reference**
***Immune checkpoint inhibitors***									
CTLA-4	Ipi	–	73	M	PR	–	–	2023	*Mathavan*
PD-1	Nivo	PD-L1 scr ≥1% + MSI	–	–	SD	–	–	2020	*Jia Z*
PD-1	Nivo	MSI high	–	–	SD	–	–	2020	*Jia Z*
PD-1	Nivo	TMB 3.5 Mut/Mb	61	F	SD	19 m	–	2022	*Almassi*
PD-1	Tislelizumab	–	62	M	SD	4 m	–	2021	*Zheng*
PD-1	Tislelizumab	TMB high	–	–	SD	5.2 m	>5.2 m	2021	*Chen*
PD-1	Toripalimab	TMB high	–	–	SD	>6.5 m	>6.5 m	2021	*Chen*
PD-1	Pembrolizumab	–	47	F	PD	–	27.7 m	2023	*Mathavan*
PD-1	Nivo	–	73	M	PD	–	–	2023	*Mathavan*
PD-1	Pembrolizumab	–	56	M	PR	–	21.7 m	2023	*Mathavan*
PD-1	Pembrolizumab	MSI high	75	F	SD	5 cycles	–	2022	*Tokita*
PD-1, CTLA-4	Nivo+Ipi	–	–	–	PR	–	–	2020	*McGregor*
PD-1, CTLA-4	Nivo+Ipi	–	–	–	SD	–	–	2020	*McGregor*
PD-1, CTLA-4	Nivo+Ipi	–	–	–	SD	–	–	2020	*McGregor*
PD-1, CTLA-4	Nivo+Ipi	–	–	–	PD	–	–	2020	*McGregor*
PD-1, CTLA-4	Nivo+Ipi	–	73	M	PD	–	–	2023	*Mathavan*
PD-L1	Atezolizumab	*MSH6 mut.MSH6 mut.*	–	–	SD	21 w	>21 w	2017	*Kardos*
PD-L1	Atezolizumab	PD-L1 exp., TMB high	73	M	SD	–	–	2023	*Mathavan*
					**ORR: 3/18 =17%**				
***Tyrosine kinase inhibitors***									
EGFR	Gefitinib	EGFR overexp.	–	–	PR	<4 w	–	2005	*Goss*
EGFR	Cetuximab	*EGFR amp.EGFR amp.*	35	M	PR	>8 m	>8 m	2016	*Collazo-Lorduy*
EGFR	Cetuximab	*EGFR mut.EGFR mut.*	46	M	SD	15 m	PFS>15 m	2012	*Wessendorf*
HER2	Neratinib	–	47	F	SD	4 m	27.7 m	2023	*Mathavan*
MEK	Trametinib	*KRAS, GNAS mut.KRAS, GNAS mut.*	42	F	–	–	–	2016	*Loh*
MEK	Trametinib	*MAP2K1 mut.MAP2K1 mut.*	60	F	SD	10 m	–	2016	*Loh*
MEK	Trametinib	*KRAS (Q61L) mut.KRAS (Q61L) mut.*	61	F	SD	30 m	–	2022	*Almassi*
MET	Tepotinib	–	59	M	–	–	PFS:12.9 m	2020	*Shitara*
RTK	Sunitinib	–	33	F	SD	–	5 m	2014	*Testa*
RTK	Sorafenib	*FLT3 amp.*	60	F	–	4 w	–	2016	*Loh*
RTK	Sunitinib	*MAP2K1(p.K57N) mut.*	60	F	mixed resp.	–	–	2016	*Loh*
RTK	Anlotinib	–	49	M	SD	–	>4 m	2016	*Wang*
VEGFR	Afatinib	–	–	–	SD	–	–	2020	*Jia Z*
VEGFR	Bevacizumab	–	–	–	SD	–	–	2020	*Jia Z*
VEGFR	Bevacizumab	–	–	–	PD	–	–	2020	*Jia Z*
VEGFR	Bevacizumab	*KRAS (p.G12V) mut.*	62	M	PR	18 w	–	2022	*Zheng*
VEGFR	Bevacizumab	–	68	M	SD	12 m	20 m	2015	*Kanamaru*
					**ORR: 3/13 =23%**				
***PARP inhibitors***									
PARPi	Niraparib	–	–	–	PD	–	–	2021	*Yonemori*
PARPi	Rucaparib	*BRCA1 del.*	42	F	CR	19 m	>19 m	2019	*Seto*
***Antibody-drug conjugate***									
Nectin-4	Enfortumab Vedotin	–	48	M	CR	15 m	PFS>15 m	2022	*Adib*
**B) Primary Bladder Adenocarcinoma**									
**Target**	**Drug name**	**Molecular target**	**Age**	**Sex**	**Rad. resp.**	**Resp. duration**	**Survival**	**Year**	***Reference***
***Immune checkpoint inhibitors***									
PD-1	Pembrolizumab	–	–	F	–	–	>18 m	2022	*Almassi*
PD-1	Nivo	–	–	M	PD	–	–	2022	*Almassi*
PD-1	Pembrolizumab	–	–	F	–	–	–	2022	*Almassi*
					**ORR: N/A**				
***Tyrosine kinase inhibitors***									
VEGFR	Bevacizumab	EGFR overexp.	44	M	CR	13 m	13 m	2005	*Teo*
VEGFR	Bevacizumab	–	25	F	PR/SD	15 m	>15 m	2011	*Valerio*
VEGFR	Bevacizumab	N/A	54	M	PR/SD	3 m	26.4 m	2016	*Pokuri*
VEGFR	Bevacizumab	N/A	54	M	PD	N/A	26.4 m	2016	*Pokuri*
EGFR	Cetuximab	N/A	54	M	SD	N/A	26.4 m	2016	*Pokuri*
HER2	Trastuzumab	*HER2 amp./IHC+*	64	M	PR	6 m	PFS>6 m	2020	*Wang*
					**ORR: 4/6 =67%**				

Limited data are available about the role of PARP inhibitors or antibody–drug conjugates (ADCs). Only two UrC patients received PARP inhibitor treatment; one patient with unknown homologous recombination repairstatus had a partial response to niraparib, while the other patient harbouring a *BRCA1* deletion benefited by a complete response to rucaparib treatment. In addition, one publication reported an UrC patient who had a complete response on enfortumab vedotin (EV) [35, 78].

Less data is available for PBAC (Table 1). Despite treatment attempts with PD-1 inhibitors no survival data has been reported to date [79]. Notably, PBAC had higher ORR compared to UrC for tyrosine kinase inhibitors (67% *vs.* 23%). In addition, bevacizumab treatment resulted in a complete response in pulmonary metastases in one PBAC patient with EGFR overexpression [39]. A patient with PBAC and *ERBB2* (HER2) amplification had partial response to anti-HER2 trastuzumab therapy [46].

## Discussion

In this review, we created a comprehensive genetic library of cancers related to the urinary bladder with glandular morphology to provide a molecular framework for differential diagnosis and to support clinicians in selecting potential therapeutic targets. Such a comprehensive review is currently lacking. Furthermore, we catalogued all published clinical experience on targeted treatments for both UrC and PBAC to date to help prioritizing potentially effective off-label treatments, aiming to optimize therapeutic decision-making. Using OncoPrints, we presented and compared the top 30 mutated genes in all three tumor types (UrC, PCBAC, UCg). To gain functional insights, we highlighted the most frequently affected oncogenic signaling pathways. In addition, we evaluated the potential implications of PD-L1 and MSI status for the efficacy of ICI treatment in these tumor types.

From the top 30 most frequently altered genes in UrC, PBAC and UCg, we demonstrate that alterations in *TP53, KRAS*, *SMAD4, MYC*, *APC* and *ARID1A* were overlapping (Figs. 1 and 2).

*TP53* is frequently affected in various tumor types. Our analysis accordingly revealed similarly high frequencies (74%−79%) in all three tumor types, mostly being missense mutations (Fig. S2) [80]. p53 is a central element of the cell cycle pathway (Fig. 3A). It has been widely regarded as an undruggable target. However, alternative strategies are emerging *e.g.* blocking the interactions of p53 with its inhibitors in addition to novel gene therapy approaches [81]. An effective targeted therapy (currently ongoing clinical trials) for p53 could impact multiple types of cancers [82], including the three analyzed tumor types in the present study.

K-Ras is a small G-protein regulating cell growth and APC is an element of β-catenin destruction complex in the Wnt signaling pathway often affected by mutations in CRC [83]. Albeit these genes are altered in all the three tumor types (and UC as well), it has been proposed that UrC and CRC, due to similar histological appearance, show overlapping genetic characteristics [62, 63]. Notably, several components of the Wnt pathway have been altered in both UrC and PBAC (Fig. 3C). The key effector molecule of this pathway, β-catenin, exhibits a shift from its normal membrane and cytoplasmic localization to the nucleus, where it functions as a transcriptional coregulator (Table S3.). In our analysis, surprisingly, only five of the genes (including *KRAS* and *APC*) were shared between the top 30 altered genes of UrC and CRC. However, *APC* is affected with a much higher frequency in CRC (73%) compared to UrC (10%) and PBAC (8%), therefore *APC* alterations may help to differentiate between bladder adenocarcinomas (UrC/PBAC) and CRC invasion to the urinary bladder. Targeted treatment based on *KRAS* mutations showed promising response in UrC [11, 84]. The K-Ras specific inhibitor antibodies sotorasib and adagrasib have been approved for non-small cell lung cancer with *KRAS*^*G12C*^ mutations [77, 85]. As *KRAS* alterations are frequent also in UrC (34%) and PBAC (17%) we also assessed the occurrence of the *KRAS*^*G12C*^ mutations and found this particular alteration to be extremely rare both in UrC (1/291), and in PBAC (1/214). Despite its rarity, *KRAS*^*G12C*^ -targeting agents might represent a therapeutic option for these tumors based on a biological rationale. In addition, several clinical trials targeting non-G12C *KRAS* muatations are underway in different tumor types, thus making *KRAS* mutations in UrC and PBAC a potential therapeutic target in the future [86].

*ARID1A* is one of the subunits of the SWI/SNF chromatin remodeling complex, relatively frequently altered in all malignancies (~ 6%), especially affecting ovarian cancers and uterine endometrioid carcinoma [87]. *ARID1A* mutations (in 8% and 11% of UrC and PBAC cases, respectively) are associated with longer progression-free survival after ICI treatment [88] and mutations may contribute to resistance against platinum-based chemotherapy [89].

*PIK3CA* encodes the alpha-subunit of phosphatidyl-3-kinase, a key enzyme in the regulation of cell growth, differentiation and motility [90]. Mutations frequently occur in hormone receptor positive and HER2 negative breast cancer. Targeted therapies such as alpelisib or capivasertib for *PIK3CA* mutated patients already are available for metastatic breast cancer patients [91, 92]. As *PIK3CA* mutations are present in 7–9% of the three analyzed tumor types, these treatments might be effective. Our targeted treatment search did not identify any clinically documented experience in UrC and PBAC patients, thus the efficacy in PBAC and UrC remains unknown [93].

Apart from the therapeutic aspect, we also adressed the differential diagnostic value of the distribution of different mutations between the tumor types. Our analysis showed that PBAC on the mutational level has the highest similarity to UCg with 67% of the top affected genes being identical. Interestingly, CRC shared only 17% overlap with UrC, whereas UrC and PBAC had a considerable 50% overlap in their top 30 affected genes. These shared genetic characteristics suggest similar pathogenic mechanisms and the potential for overlapping efficacy of targeted therapies.

The considerable overlap between the mutational patterns of UrC and PBAC makes their differentiation difficult even at the genetic level. Only the different mutational frequency of *DICER* (UrC: 15% PBAC: 2%) and *KRAS* (UrC: 34, PBAC: 17%) may provide some differential diagnostic value. *DICER* encodes a ribonuclease enzyme that generates mature miRNA [94] and is known to act as a tumor suppressor. Similarly, *LPRB1* and *GNAS* alterations are more frequent in UrC compared to PBAC and thus may have some value to be included in a differential diagnostic panel.

Our analyses revealed high frequencies of *TERT* promoter mutations in UC (74%) and UCg (67%) [95], in contrast to UrC (2%) and PBAC (14%). This suggests the mutations of *TERT* promoter as potential differential diagnostic markers for these tumor types [96]. **Notably, the presence of a wild-type TERT promoter was associated with a higher objective response rate **^**64**^. Because glandular differentiation is common in UC, it is possible that at least some of the tumors reported as bladder adenocarcinoma that harbor *TERT* promoter mutations may in fact be UCs with extensive glandular differentiation in which the urothelial component was not identified. This may be compounded by the fact that only the parts of the tumor with glandular morphology are submitted for molecular analysis without central pathology review of all available material from the tumor. Supporting this argument is the following, 1) the majority of the reported bladder adenocarcinoma cases harboring *TERT* promoter mutation also harbor many of the mutations that are commonly associated with UC (*RB1, ARID1A, CDKN2A/B* loss), 2) in these tumors, reported *TERT* promoter mutations are virtually mutually exclusive with *KRAS* or *SMAD4* mutations, which are more typical of a true adenocarcinoma in this setting, and 3) none of the reported UrC harbor *TERT* promoter mutation, which is most likely related to the fact that in the urachal location the vast majority of tumors (> 90%) are pure adenocarcinoma and the possibility of UCg is exceedingly rare.

Treatment of metastatic UC with ICIs, like pembrolizumab, atezolizumab and nivolumab showed significant clinical benefits in multiple clinical trials [97]. Improved efficacy of ICI treatments were often shown to be associated with high expression of PD-L1 as well as with MSI-high and TMB-high phenotypes [98, 99]. As being the threshold for pembrolizumab companion diagnostic testing in the first line setting for advanced UC, the PD-L1 related score of CPS (combined positivity score) with a value of ≥ 10 is present in 56% of pure UC cases [100]. Histological subtypes of UC, however, also express moderate levels of PD-L1[101, 102]. Our analyses showed lower rates of high PD-L1 expression in UrC (16%) and PBAC (7%) and generally low occurrence of MSI and low TMB levels, suggesting a lower efficacy of ICIs in the three analyzed tumor types. Accordingly, available data on ICI treatment in UrC patients (n = 18) (Table 1.) showed a moderate ORR (17%) and a considerable DCR (78%), which however decreased to 64% when excluding seven patients with TMB-high or MSI-high phenotypes (all having stable disease as radiographic response). Also in PBAC (Table 1.), although the data is limited, pembrolizumab treatment resulted in over 18 months of survival in one case among the three reported patients. Based on these experiences, despite low frequency, the determination of TMB and PD-L1 in UrC might provide some therapeutic guidance.

EV is a nectin-4 targeting ADC approved for the treatment of locally advanced or metastatic UC [103]. For UrC the only published case treated with EV showed a complete response [78]. Accordingly, a former study showed high nectin-4 expression rates of 82% of bladder adenocarcinomas (including UrC cases) [101].

In another approach, we investigated how many of the top 30 mutated genes have been reported to be targeted by specific therapies in either UrC or PBAC. Only 2 of the top 30 affected genes have been targeted previously in both tumor types (*KRAS* in both, *EGFR* in UrC and *ERBB2* in PBAC). In UrC *EGFR* showed alterations (predominantly amplification) in 5% cases and two cases were reported to be treated with respective targeted therapies. Gefitinib was administered in a phase I clinical trial with various solid tumors including lung, breast, colon, cervix and ovarian cancers along with one case of UrC with lymphatic metastasis. From the 28 included patients, four showed clinical evidence of response and the UrC case showed the greatest reduction of tumor size (55%). This considerable partial remission was accompanied by a biological response as shown by a moderate decrease of Ki67 proliferation index in the post treatment tumor biopsy [52]. In addition, one metastatic UrC patient with *EGFR* amplification showed an eight-month partial response after failure of two lines of chemotherapy [104]. These findings highlight EGFR as a potential target in UrC. Regarding PBAC, *ERBB2* (HER2) is one of the most altered genes that has been targeted with a specific inhibitor, trastuzumab. This case showed progression-free survival for more than 6 months [46]. In addition, the HER2-targeting antibody–drug conjugate trastuzumab deruxtecan received tumor-agnostic FDA-approval for patients with HER2-overexpressing metastatic tumors who have progressed on after at least one previous line of therapy or have no available standard treatment [105]. Notably, in the targeted treatment of PBAC the sequencing of *ERBB2* may have a key role in selecting efficient targeted approaches based on its relatively common mutations, similar to the approach used in non-small cell lung cancer (NSCLC) [106].

From the top 30 mutated genes, further candidates can be highlighted as potential predictors of targeted treatments. *LRP1B* is frequently (11%) affected in UrC. It has been suggested as a prognostic marker in gastric cancer. In addition, *LRP1B* mutations were shown to be associated with better outcomes in ICI treated patients [107].

Furthermore, *FLT3* (a tyrosin kinase receptor with 6% alteration rate in PBAC) alterations have FDA-approved inhibitory antibodies are available in AML and it might be a favorable target in PBAC [108]. *MET* is altered in 3% of PBAC patients through amplifications. It encodes an RTK and targeted therapies for *MET* amplifications have been approved [109]. *MET* amplification can lead to resistance to anti-EGFR targeted treatment as well [110]. Furthermore, the FDA and EMA approved agents capmatinib and tepotinib are applied in NSCLC with *MET* exon14 skipping mutations [46]. This leads to a truncated protein and is associated with an activated HGF/MET pathway. As this alteration is usually detected by RNA-based technologies, only one study analyzed this alteration in UrC and PBAC revealing a relatively high occurrence of 10% in both tumor types [10]. Small molecules specific to *MET* are also in development and have been tested in patients with solid tumors including bladder malignancies [111].

The rarity of these malignancies will remain a significant barrier to gathering high-quality clinical evidence. Therefore, it is crucial to continuously collect clinical data from individual cases across multiple institutions. To facilitate such efforts, we aim to create a publicly accessible repository based on the collection of genetic data and targeted treatment experiences (urachalcancer.org) – including the currently summarized and evaluated dataset – and will be continuously updated by novel published information. By making the raw data of the OncoPrints as downloadable supplementary data, we encourage other investigators, clinicians, and geneticists to further study and investigate gene associations and integrate with their own data and generate hypotheses for basic science research projects.

This study has some important limitations. **The included studies used different sequencing platforms, had incomplete genetic panel coverage and varying data evaluation methods that could not be adjusted retrospectively. Therefore, a meta-analytic or comparative statistical testing was not feasible. Our comparisons do not include inferential statistics; the statistical analysis is solely descriptive due to heterogeneous sequencing platforms, incomplete gene coverage. The high-throughput nature of recent NGS studies may introduce selection bias toward more recent, predominantly NGS-based studies, potentially limiting the representativeness of earlier literature**. In addition, the **variable** reporting of molecular characteristics (TMB, PD-L1 positivity, β-catenin) might have had different thresholds and cut-offs, and **no centralized pathological reviews were carried out**. **In addition, our evaluation was based on neither prospective nor randomized datasets**. Publications reporting targeted treatment experiences were **based on small case number studies with heterogeneous response criteria and follow-up** and may be biased towards cases with better results. **Therefore, the derived ORR and DCR results should be considered exploratory rather than robust estimates of efficacy**. The strengths of this study lie in its comprehensive nature providing an up-to-date overview of our current knowledge on the understudied field of rare bladder associated carcinomas with glandular morphology. We believe that this integrated dataset can also serve as a foundation for the selection of novel and effective targeted therapies. In addition, we catalogued all previous attempts of targeted treatments in UrC and PBAC. By bringing together these datasets, clinicians have a theoretical basis to choose potentially effective personalized treatment options for UrC and PBAC.

## Supplementary Information

Below is the link to the electronic supplementary material.

ESM 1 (XLSX 17.8 KB)

ESM 2 (XLSX 1.78 MB)

ESM 3 (XLSX 13.5 KB)

ESM 4 (XLSX 23.4 KB)

ESM 5 (PNG 766 KB)

(AI 5.37 MB)

ESM 6 (PNG 1.39 MB)

(AI 7.47 MB)

## Data Availability

No datasets were generated or analysed during the current study.

## Abbreviations

ADC: Antibody-drug conjugates
CIS: Urothelial carcinoma in situ
CRC: Colorectal carcinoma
DCR: Disease control rate
EV: Enfortumab vedotin
ICI: Immune checkpoint inhibitor
MMR: Mismatch repair
MSI: Microsatellite instability
NSCLC: Non-small cell lung cancer
UC: Urothelial carcinoma
UrC: Urachal carcinoma
UCg: Urothelial carcinoma with glandular differentiation
ORR: Objective/overall response rate
PBAC: Primary bladder adenocarcinomas
RTK: Receptor tyrosine kinase
TMB: Tumor mutational burden
