# Supplementary figures and images for: Genetic characteristics and targeted treatments of primary bladder and urachal adenocarcinomas: a systematic review with pooled descriptive genomic analyses
Source: Cancer Metastasis Rev. 2026 May 1;45(2):26. doi: 10.1007/s10555-026-10332-3 (PMC13133215; doi:10.1007/s10555-026-10332-3)

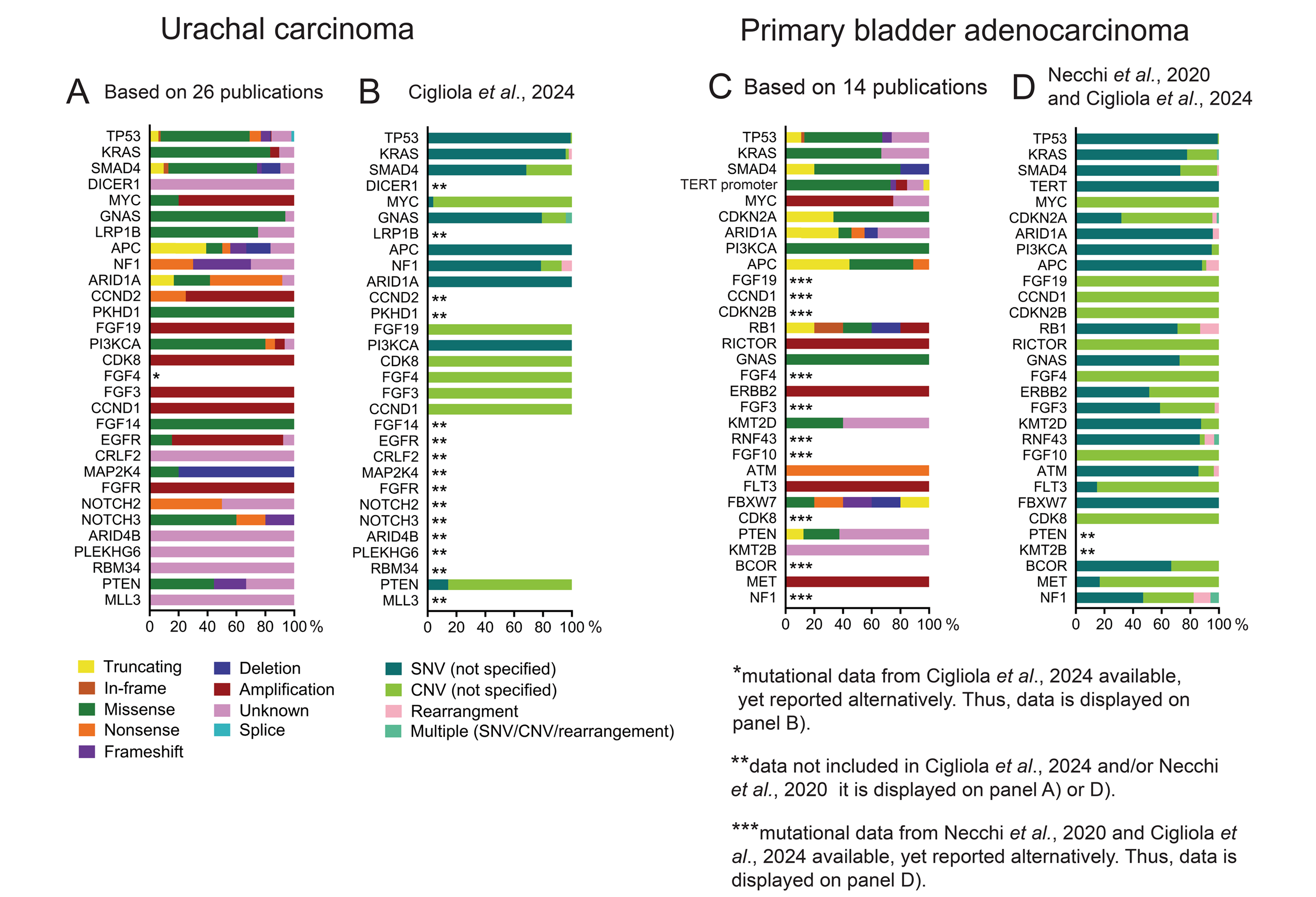

Supplement: Supplementary file 5 — (PNG 766 KB) [file 10555_2026_10332_Fig4_ESM.png]

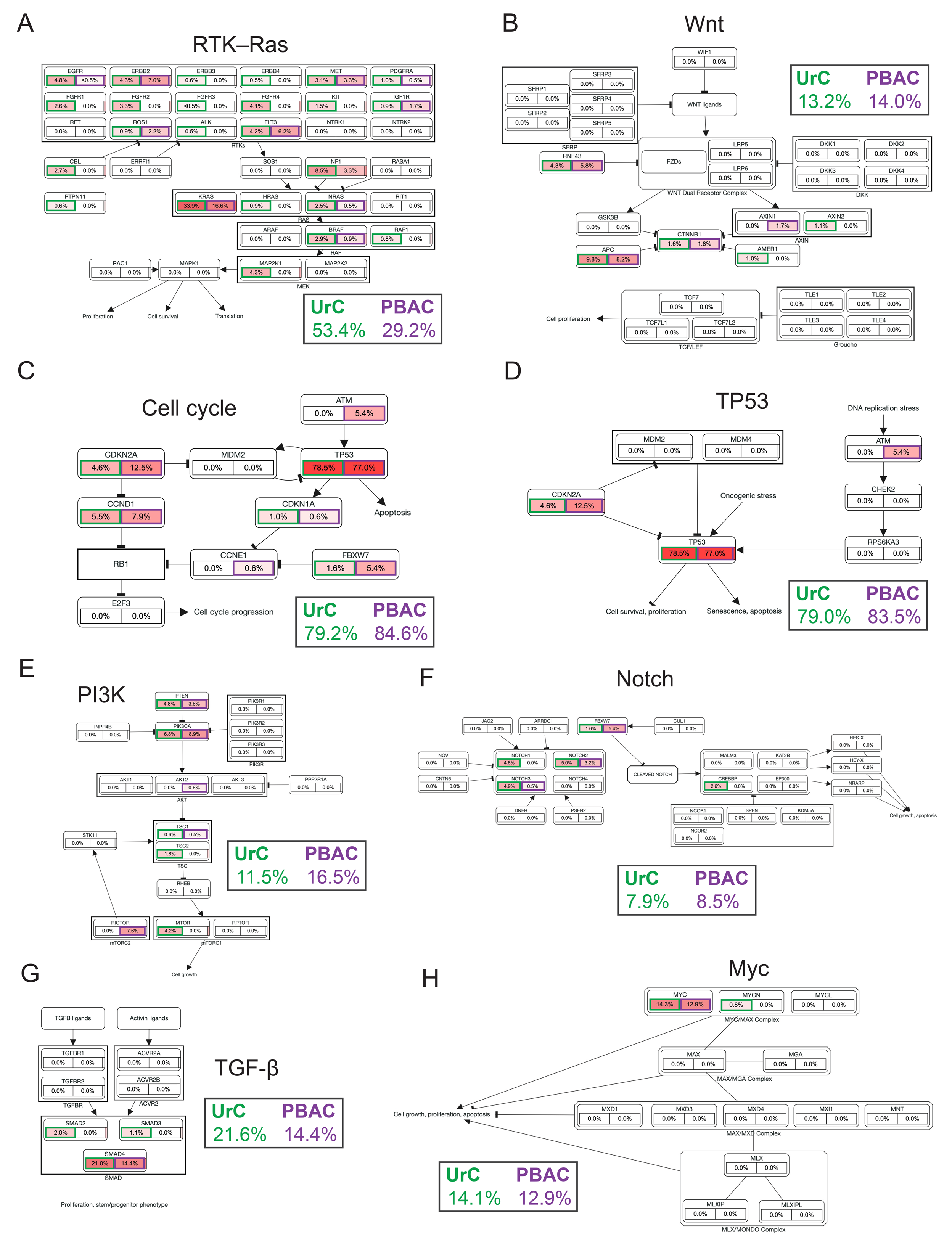

Supplement: Supplementary file 7 — (PNG 1.39 MB) [file 10555_2026_10332_Fig5_ESM.png]
